# An extended Hilbert’s integral inequality in the whole plane with parameters

**DOI:** 10.1186/s13660-018-1810-z

**Published:** 2018-08-20

**Authors:** Leping He, Yin Li, Bicheng Yang

**Affiliations:** 1 College of Mathematics and Statistics, Jishou University, Jishou, P.R. China; 2 Department of Mathematics, Guangdong University of Education, Guangzhou, P.R. China

**Keywords:** 26D15, 47A05, Hilbert’s integral inequality, Weight function, Equivalent form, Operator, Norm

## Abstract

By introducing independent parameters and interval variables, applying the weight functions and the technique of real analysis, an extended Hilbert’s integral inequality in the whole plane with parameters and a best possible constant factor is provided. The equivalent forms, the reverses, and the related homogeneous forms with particular parameters are considered. Meanwhile, an extended Hilbert’s integral operator in the whole plane is defined, and the operator expressions for the equivalent inequalities are obtained.

## Introduction

Assuming that $0 < \int_{0}^{\infty} f^{2}(x) \,dx < \infty $ and $0 < \int_{0}^{\infty} g^{2}(y) \,dy < \infty $, we have the following well-known Hilbert’s integral inequality with the best possible constant factor *π* [1]:

1 $$ \int_{0}^{\infty} \int_{0}^{\infty} \frac{f(x)g(y)}{x + y} \,dx \,dy < \pi \biggl( \int_{0}^{\infty} f^{2}(x) \,dx \int_{0}^{\infty} g^{2}(y) \,dy \biggr)^{\frac{1}{2}}. $$

In 1925, Hardy gave an extension of (1) as follows [2]: If $p > 1$, $\frac{1}{p} + \frac{1}{q} = 1 $, $f(x) \ge 0$, satisfying $0 < \int_{0}^{\infty} f^{p}(x) \,dx < \infty $, and $g(y) \ge 0 $, satisfying $0 < \int_{0}^{\infty} g^{q}(y) \,dy < \infty $, then we have

2 $$ \int_{0}^{\infty} \int_{0}^{\infty} \frac{f(x)g(y)}{x + y} \,dx \,dy < \frac{\pi}{\sin (\frac{\pi}{p})}\biggl( \int_{0}^{\infty} f^{p}(x) \,dx \biggr)^{\frac{1}{p}}\biggl( \int_{0}^{\infty} g^{q}(y) \,dy \biggr)^{\frac{1}{q}}, $$

where the constant factor $\pi /\sin (\frac{\pi}{p}) $ is still the best possible. We call (2) Hardy–Hilbert’s integral inequality, which with (1) is important in analysis and its applications (cf. [1, 3]). In 1934, Hardy et al. gave an extension of (2) with the general homogeneous kernel of degree −1 (see [1], Theorem 319). Meanwhile, a Hilbert-type integral inequality with the general nonhomogeneous kernel is provided (see [1], Theorem 350): If $h(x) > 0$, $\int_{0}^{\infty} h(x)x^{s - 1} \,dx = \phi (s) \in \textbf{R}_{ +} = (0,\infty ) $, then

3 $$ \int_{0}^{\infty} \int_{0}^{\infty} h(xy)f(x)g(y) \,dx \,dy < \phi \biggl( \frac{1}{p}\biggr) \biggl( \int_{0}^{\infty} x^{p - 2}f^{p}(x) \,dx \biggr)^{\frac{1}{p}}\biggl( \int_{0}^{\infty} g^{q}(y) \,dy \biggr)^{\frac{1}{q}}. $$

By introducing an independent parameter $\lambda \in (0,\infty ) $ and the beta function, in 1998, Yang [4] gave an extension of (1) as follows:

4 $$ \int_{0}^{\infty} \int_{0}^{\infty} \frac{f(x)g(y)}{(x + y)^{\lambda}} \,dx \,dy < B\biggl( \frac{\lambda}{2},\frac{\lambda}{2}\biggr) \biggl( \int_{0}^{\infty} x^{1 - \lambda} f^{2}(x) \,dx \int_{0}^{\infty} y^{1 - \lambda} g^{2}(y) \,dy\biggr)^{\frac{1}{2}}, $$

where the constant factor $B(\frac{\lambda}{2},\frac{\lambda}{2}) $ is the best possible, and

$$B(u,v): = \int_{0}^{\infty} \frac{t^{v - 1}}{(1 + t)^{u + v}} \,dt\quad (u,v > 0) $$

is the beta function (cf. [5]).

In 2007, Li [6] gave an extension of (4) and Yang [7] provided the following Hilbert-type integral inequality with the nonhomogeneous kernel:

5 $$ \int_{0}^{\infty} \int_{0}^{\infty} \frac{f(x)g(y)}{1 + (xy)^{\lambda}} \,dx \,dy < \frac{\pi}{\lambda} \biggl( \int_{0}^{\infty} x^{p(1 - \frac{\lambda}{2}) - 1}f^{p}(x) \,dx \biggr)^{\frac{1}{p}}\biggl( \int_{0}^{\infty} y^{q(1 - \frac{\lambda}{2}) - 1}g^{q}(y) \,dy \biggr)^{\frac{1}{q}}. $$

Since then, a lot of authors have continued to discuss this topic (cf. [8–14]).

In this paper, by introducing independent parameters and interval variables, applying the weight functions and the technique of real analysis, a Hilbert-type integral inequality in the whole plane with parameters and a best possible constant factor is provided as follows:

6 $$ \begin{aligned}[b] & \int_{ - \infty}^{\infty} \int_{ - \infty}^{\infty} \frac{f(x)g(y)}{( \vert x \vert + \vert y \vert )^{\lambda}} \,dx \,dy \\ &\quad < 2B(\mu,\sigma )\biggl[ \int_{ - \infty}^{\infty} \vert x \vert ^{p(1 - \mu ) - 1} f^{p}(x)\,dx\biggr]^{\frac{1}{p}}\biggl[ \int_{ - \infty}^{\infty} \vert y \vert ^{q(1 - \sigma ) - 1} g^{q}(y)\,dy\biggr]^{\frac{1}{q}} \end{aligned} $$

($\mu,\sigma > 0$, $\mu + \sigma = \lambda $), which is an extension of (4). The more general form of (6) with parameters, the equivalent inequalities, the reverses, and the related homogeneous form with the particular parameter are considered. Meanwhile, an extended Hilbert’s integral operator in the whole plane is defined, and the operator expressions for the equivalent inequalities are obtained.

## Weight functions and an initial inequality

### Definition 1

Suppose that $\delta \in \{ - 1,1\} $, $- 1 < \alpha,\beta < 1 $, $\mu,\sigma > 0$, $\mu + \sigma = \lambda $. Define the following weight functions:

7 $$\begin{aligned}& \omega_{\delta} (\sigma,y): = \bigl( \vert y \vert + \beta y \bigr)^{\sigma} \int_{ - \infty}^{\infty} \frac{( \vert x \vert + \alpha x)^{\delta \sigma - 1}}{[1 + ( \vert x \vert + \alpha x)^{\delta} ( \vert y \vert + \beta y)]^{\lambda}} \,dx\quad \bigl(y \in \textbf{R} = ( - \infty,\infty )\bigr), \end{aligned}$$

8 $$\begin{aligned}& \varpi_{\delta} (\sigma,x): = \bigl( \vert x \vert + \alpha x \bigr)^{\delta \sigma} \int_{ - \infty}^{\infty} \frac{( \vert y \vert + \beta y)^{\sigma - 1}}{[1 + ( \vert x \vert + \alpha x)^{\delta} ( \vert y \vert + \beta y)]^{\lambda}} \,dy\quad (x \in \textbf{R}). \end{aligned}$$

We find

$$\begin{aligned} &\omega_{\delta} (\sigma,y)\\ &\quad = \bigl( \vert y \vert + \beta y\bigr)^{\sigma} \biggl\{ \int_{ - \infty}^{0} \frac{( - x + \alpha x)^{\delta \sigma - 1}\,dx}{[1 + ( - x + \alpha x)^{\delta} ( \vert y \vert + \beta y)]^{\lambda}} + \int_{0}^{\infty} \frac{(x + \alpha x)^{\delta \sigma - 1}\,dx}{[1 + (x + \alpha x)^{\delta} ( \vert y \vert + \beta y)]^{\lambda}} \biggr\} \\ &\quad = \bigl( \vert y \vert + \beta y\bigr)^{\sigma} \biggl\{ \int_{0}^{\infty} \frac{(x - \alpha x)^{\delta \sigma - 1}\,dx}{[1 + (x - \alpha x)^{\delta} ( \vert y \vert + \beta y)]^{\lambda}} + \int_{0}^{\infty} \frac{(x + \alpha x)^{\delta \sigma - 1}\,dx}{[1 + (x + \alpha x)^{\delta} ( \vert y \vert + \beta y)]^{\lambda}} \biggr\} . \end{aligned} $$

For fixed *y* (≠0), setting $u = (x - \alpha x)^{\delta} (|y| + \beta y) $ in the above first integral, we obtain that $x = \frac{(|y| + \beta y)^{ - \frac{1}{\delta}}}{1 - \alpha} u^{\frac{1}{\delta}}$, $dx = \frac{(|y| + \beta y)^{ - \frac{1}{\delta}}}{\delta (1 - \alpha )}u^{\frac{1}{\delta} - 1}\,du $, and

$$\begin{aligned} \int_{0}^{\infty} \frac{(x - \alpha x)^{\delta \sigma - 1}\,dx}{[1 + (x - \alpha x)^{\delta} ( \vert y \vert + \beta y)]^{\lambda}} &= \int_{0}^{\infty} \frac{u^{\sigma - \frac{1}{\delta}}}{(1 + u)^{\lambda} ( \vert y \vert + \beta y)^{\sigma - \frac{1}{\delta}}} \frac{u^{\frac{1}{\delta} - 1}}{(1 - \alpha )( \vert y \vert + \beta y)^{\frac{1}{\delta}}} \,du \\ &= \frac{1}{(1 - \alpha )( \vert y \vert + \beta y)^{\sigma}} \int_{0}^{\infty} \frac{u^{\sigma - 1}}{(1 + u)^{\lambda}} \,du. \end{aligned} $$

In the same way, setting $u = (x + \alpha x)^{\delta} (|y| + \beta y) $ in the above second integral, it follows that

$$\int_{0}^{\infty} \frac{(x + \alpha x)^{\delta \sigma - 1}\,dx}{[1 + (x + \alpha x)^{\delta} ( \vert y \vert + \beta y)]^{\lambda}} = \frac{1}{(1 + \alpha )( \vert y \vert + \beta y)^{\sigma}} \int_{0}^{\infty} \frac{u^{\sigma - 1}}{(1 + u)^{\lambda}} \,du. $$

Hence, we have

9 $$ \omega_{\delta} (\sigma,y) = \biggl(\frac{1}{1 - \alpha} + \frac{1}{1 + \alpha} \biggr) \int_{0}^{\infty} \frac{u^{\sigma - 1}}{(1 + u)^{\lambda}} \,du = K_{\alpha} (\sigma ): = \frac{2}{1 - \alpha^{2}}B(\mu,\sigma ). $$

By (8), we find

$$\begin{aligned} &\varpi_{\delta} (\sigma,x)\\ &\quad = \bigl( \vert x \vert + \alpha x\bigr)^{\delta \sigma} \biggl\{ \int_{ - \infty}^{0} \frac{( - y + \beta y)^{\sigma - 1}\,dy}{[1 + ( \vert x \vert + \alpha x)^{\delta} ( - y + \beta y)]^{\lambda}} + \int_{0}^{\infty} \frac{(y + \beta y)^{\sigma - 1}\,dy}{[1 + ( \vert x \vert + \alpha x)^{\delta} (y + \beta y)]^{\lambda}} \biggr\} \\ &\quad = \bigl( \vert x \vert + \alpha x\bigr)^{\delta \sigma} \biggl\{ \int_{0}^{\infty} \frac{(y - \beta y)^{\sigma - 1}\,dy}{[1 + ( \vert x \vert + \alpha x)^{\delta} (y - \beta y)]^{\lambda}} + \int_{0}^{\infty} \frac{(y + \beta y)^{\sigma - 1}\,dy}{[1 + ( \vert x \vert + \alpha x)^{\delta} (y + \beta y)]^{\lambda}} \biggr\} . \end{aligned} $$

For fixed *x* (≠0), setting $u = (|x| + \alpha x)^{\delta} (1 - \beta )y $ in the above first integral, we obtain $y = \frac{u}{(1 - \beta )(|x| + \alpha x)^{\delta}}$, and

$$\begin{aligned} \int_{0}^{\infty} \frac{(y - \beta y)^{\sigma - 1}\,dy}{[1 + ( \vert x \vert + \alpha x)^{\delta} (y - \beta y)]^{\lambda}} &= \int_{0}^{\infty} \frac{u^{\sigma - 1}}{(1 + u)^{\lambda} ( \vert x \vert + \alpha x)^{\delta (\sigma - 1)}} \frac{du}{(1 - \beta )( \vert x \vert + \alpha x)^{\delta}} \\ &= \frac{1}{(1 - \beta )( \vert x \vert + \alpha x)^{\delta \sigma}} \int_{0}^{\infty} \frac{u^{\sigma - 1}}{(1 + u)^{\lambda}} \,du. \end{aligned} $$

In the same way, setting $u = (|x| + \alpha x)^{\delta} (1 + \beta )y $ in the above second integral, we find

$$\int_{0}^{\infty} \frac{(y + \beta y)^{\sigma - 1}\,dy}{[1 + ( \vert x \vert + \alpha x)^{\delta} (y + \beta y)]^{\lambda}} = \frac{1}{(1 + \beta )( \vert x \vert + \alpha x)^{\delta \sigma}} \int_{0}^{\infty} \frac{u^{\sigma - 1}}{(1 + u)^{\lambda}} \,du, $$

and then

10 $$ \varpi_{\delta} (\sigma,x) = \biggl(\frac{1}{1 - \beta} + \frac{1}{1 + \beta} \biggr) \int_{0}^{\infty} \frac{u^{\sigma - 1}}{(1 + u)^{\lambda}} \,du = K_{\beta} (\sigma ): = \frac{2}{1 - \beta^{2}}B(\mu,\sigma ). $$

### Theorem 1

*Suppose that* $p > 0$ ($p \ne 1$), $\frac{1}{p} + \frac{1}{q} = 1$, $\delta \in \{ - 1,1\}$, $- 1 < \alpha,\beta < 1 $, $\mu,\sigma > 0$, $\mu + \sigma = \lambda $, *and*

11 $$ K(\sigma ): = K_{\beta}^{\frac{1}{p}}(\sigma )K_{\alpha}^{\frac{1}{q}}( \sigma ) = \frac{2B(\mu,\sigma )}{(1 - \beta^{2})^{1/p}(1 - \alpha^{2})^{1/q}}. $$

*If* $f(x) \ge 0$ ($x \in \textbf{R} $), *satisfying* $0 < \int_{ - \infty}^{\infty} (|x| + \alpha x)^{p(1 - \delta \sigma ) - 1} f^{p}(x)\,dx < \infty $, *then*

(i) *for* $p > 1 $, *we have the following inequality*:
  12 $$ \begin{aligned}[b] J&: = \biggl\{ \int_{ - \infty}^{\infty} \bigl( \vert y \vert + \beta y \bigr)^{p\sigma - 1}\biggl[ \int_{ - \infty}^{\infty} \frac{f(x)}{[1 + ( \vert x \vert + \alpha x)^{\delta} ( \vert y \vert + \beta y)]^{\lambda}} \,dx \biggr]^{p}\,dy\biggr\} ^{\frac{1}{p}} \\ &< \frac{2B(\mu,\sigma )}{(1 - \beta^{2})^{1/p}(1 - \alpha^{2})^{1/q}}\biggl[ \int_{ - \infty}^{\infty} \bigl( \vert x \vert + \alpha x \bigr)^{p(1 - \delta \sigma ) - 1} f^{p}(x)\,dx\biggr]^{\frac{1}{p}}; \end{aligned} $$
(ii) *for* $0 < p < 1 $, *we have the reverse of* (12).

### Proof

(i) For $p > 1 $, by Hölder’s inequality with weight [15] and (7), when $y \ne 0 $, we find

13 $$ \begin{gathered}[b] \int_{ - \infty}^{\infty} \frac{f(x)}{[1 + ( \vert x \vert + \alpha x)^{\delta} ( \vert y \vert + \beta y)]^{\lambda}} \,dx \\ \quad = \int_{ - \infty}^{\infty} \frac{1}{[1 + ( \vert x \vert + \alpha x)^{\delta} ( \vert y \vert + \beta y)]^{\lambda}} \biggl[ \frac{( \vert x \vert + \alpha x)^{(1 - \delta \sigma )/q}}{( \vert y \vert + \beta y)^{(1 - \sigma )/p}}f(x)\biggr] \biggl[\frac{( \vert y \vert + \beta y)^{(1 - \sigma )/p}}{( \vert x \vert + \alpha x)^{(1 - \delta \sigma )/q}}\biggr]\,dx\hspace{-20pt} \\ \quad \le \biggl\{ \int_{ - \infty}^{\infty} \frac{( \vert x \vert + \alpha x)^{(1 - \delta \sigma )(p - 1)}}{[1 + ( \vert x \vert + \alpha x)^{\delta} ( \vert y \vert + \beta y)]^{\lambda}} \frac{f^{p}(x)\,dx}{( \vert y \vert + \beta y)^{1 - \sigma}} \biggr\} ^{\frac{1}{p}}\\ \qquad {}\times\biggl\{ \int_{ - \infty}^{\infty} \frac{( \vert y \vert + \beta y)^{(1 - \sigma )(q - 1)}}{[1 + ( \vert x \vert + \alpha x)^{\delta} ( \vert y \vert + \beta y)]^{\lambda}} \frac{dx}{( \vert x \vert + \alpha x)^{1 - \delta \sigma}} \biggr\} ^{\frac{1}{q}} \\ \quad = \bigl(\omega_{\delta} (\sigma,y)\bigr)^{\frac{1}{q}}\bigl( \vert y \vert + \beta y\bigr)^{\frac{1}{p} - \sigma} \biggl\{ \int_{ - \infty}^{\infty} \frac{( \vert x \vert + \alpha x)^{(1 - \delta \sigma )(p - 1)}}{[1 + ( \vert x \vert + \alpha x)^{\delta} ( \vert y \vert + \beta y)]^{\lambda}} \frac{f^{p}(x)\,dx}{( \vert y \vert + \beta y)^{1 - \sigma}} \biggr\} ^{\frac{1}{p}}.\hspace{-20pt} \end{gathered} $$

We prove that (13) takes the form of strict inequality. Otherwise, there exists $y \ne 0 $ such that (13) takes the form of equality. Then there exist constants A and B such that they are not all zero, and [15]

$$\begin{gathered} {A}\frac{( \vert x \vert + \alpha x)^{(1 - \delta \sigma )(p - 1)}}{[1 + ( \vert x \vert + \alpha x)^{\delta} ( \vert y \vert + \beta y)]^{\lambda}} \frac{f^{p}(x)}{( \vert y \vert + \beta y)^{1 - \sigma}} \\ \quad = {B}\frac{( \vert y \vert + \beta y)^{(1 - \sigma )(q - 1)}}{[1 + ( \vert x \vert + \alpha x)^{\delta} ( \vert y \vert + \beta y)]^{\lambda}} \frac{1}{( \vert x \vert + \alpha x)^{1 - \delta \sigma}}\quad \mbox{a.e. in } \textbf{R}. \end{gathered} $$

If $A = 0 $, then $B = 0 $, which is impossible. We suppose that $A \ne 0 $, namely

$$\bigl( \vert x \vert + \alpha x\bigr)^{p(1 - \delta \sigma ) - 1}f^{p}(x) = \frac{B( \vert y \vert + \beta y)^{q(1 - \sigma )}}{{A}( \vert x \vert + \alpha x)}\quad \mbox{a.e. in }\textbf{R}, $$

which contradicts the fact that $0 < \int_{ - \infty}^{\infty} (|x| + \alpha x)^{p(1 - \delta \sigma ) - 1} f^{p}(x)\,dx < \infty $.

Then by (11) and Fubini’s theorem [16], we find

14 $$ \begin{aligned}[b] J &< K_{\alpha}^{\frac{1}{q}}(\sigma )\biggl\{ \int_{ - \infty}^{\infty} \int_{ - \infty}^{\infty} \frac{( \vert x \vert + \alpha x)^{(1 - \delta \sigma )(p - 1)}}{[1 + ( \vert x \vert + \alpha x)^{\delta} ( \vert y \vert + \beta y)]^{\lambda}} \frac{1}{( \vert y \vert + \beta y)^{1 - \sigma}} f^{p}(x)\,dx \,dy\biggr\} ^{\frac{1}{p}} \\ &= K_{\alpha}^{\frac{1}{q}}(\sigma )\biggl\{ \int_{ - \infty}^{\infty} \varpi_{\delta} (\sigma,x) \bigl( \vert x \vert + \alpha x\bigr)^{p(1 - \delta \sigma ) - 1}f^{p}(x)\,dx \biggr\} ^{\frac{1}{p}}. \end{aligned} $$

In view of (10) and (11), we have (12).

(ii) For $0 < p < 1 $, by the reverse Hölder’s inequality [15], (7), and (9), we have the reverses of (13) and (14). Then, by (10) and (11), we obtain the reverse of (12).

The theorem is proved. □

## Main results

### Theorem 2

*Suppose that* $p > 1$, $\frac{1}{p} + \frac{1}{q} = 1$, $\delta \in \{ - 1,1\} $, $- 1 < \alpha,\beta < 1 $, $\mu,\sigma > 0$, $\mu + \sigma = \lambda $. *If* $f(x), g(y) \ge 0 $, *satisfying*

$$0 < \int_{ - \infty}^{\infty} \bigl( \vert x \vert + \alpha x \bigr)^{p(1 - \delta \sigma ) - 1} f^{p}(x)\,dx < \infty\quad \textit{and}\quad 0 < \int_{ - \infty}^{\infty} \bigl( \vert y \vert + \beta y \bigr)^{q(1 - \sigma ) - 1} g^{q}(y)\,dy < \infty, $$

*then we have the following inequality equivalent to* (12):

15 $$ \begin{aligned}[b] I&: = \int_{ - \infty}^{\infty} \int_{ - \infty}^{\infty} \frac{f(x)g(y)}{[1 + ( \vert x \vert + \alpha x)^{\delta} ( \vert y \vert + \beta y)]^{\lambda}} \,dx \,dy \\ &< \frac{2B(\mu,\sigma )}{(1 - \beta^{2})^{1/p}(1 - \alpha^{2})^{1/q}}\biggl[ \int_{ - \infty}^{\infty} \bigl( \vert x \vert + \alpha x \bigr)^{p(1 - \delta \sigma ) - 1} f^{p}(x)\,dx\biggr]^{\frac{1}{p}} \\ &\quad {}\times \biggl[ \int_{ - \infty}^{\infty} \bigl( \vert y \vert + \beta y \bigr)^{q(1 - \sigma ) - 1} g^{q}(y)\,dy\biggr]^{\frac{1}{q}}, \end{aligned} $$

*where the constant* $K(\sigma ) = \frac{2B(\mu,\sigma )}{(1 - \beta^{2})^{1/p}(1 - \alpha^{2})^{1/q}} $ *in* (15) *and* (12) *is the best possible*.

*In particular*, *for* $\delta = 1 $, *we have the following equivalent inequalities with the nonhomogeneous kernel and the best possible constant factor* $K(\sigma ) = \frac{2B(\mu,\sigma )}{(1 - \beta^{2})^{1/p}(1 - \alpha^{2})^{1/q}} $:

16 $$\begin{aligned}& \begin{aligned}[b] & \biggl\{ \int_{ - \infty}^{\infty} \bigl( \vert y \vert + \beta y \bigr)^{p\sigma - 1}\biggl[ \int_{ - \infty}^{\infty} \frac{f(x)}{[1 + ( \vert x \vert + \alpha x)( \vert y \vert + \beta y)]^{\lambda}} \,dx \biggr]^{p}\,dy\biggr\} ^{\frac{1}{p}} \\ &\quad < \frac{2B(\mu,\sigma )}{(1 - \beta^{2})^{1/p}(1 - \alpha^{2})^{1/q}}\biggl[ \int_{ - \infty}^{\infty} \bigl( \vert x \vert + \alpha x \bigr)^{p(1 - \sigma ) - 1} f^{p}(x)\,dx\biggr]^{\frac{1}{p}}, \end{aligned} \end{aligned}$$

17 $$\begin{aligned}& \begin{aligned}[b] & \int_{ - \infty}^{\infty} \int_{ - \infty}^{\infty} \frac{f(x)g(y)}{[1 + ( \vert x \vert + \alpha x)( \vert y \vert + \beta y)]^{\lambda}} \,dx \,dy \\ &\quad < \frac{2B(\mu,\sigma )}{(1 - \beta^{2})^{1/p}(1 - \alpha^{2})^{1/q}}\biggl[ \int_{ - \infty}^{\infty} \bigl( \vert x \vert + \alpha x \bigr)^{p(1 - \sigma ) - 1} f^{p}(x)\,dx\biggr]^{\frac{1}{p}} \\ &\qquad {}\times \biggl[ \int_{ - \infty}^{\infty} \bigl( \vert y \vert + \beta y \bigr)^{q(1 - \sigma ) - 1} g^{q}(y)\,dy\biggr]^{\frac{1}{q}}. \end{aligned} \end{aligned}$$

### Proof

By Hölder’s inequality, we find

18 $$ \begin{aligned}[b] I &= \int_{ - \infty}^{\infty} \biggl\{ \bigl( \vert y \vert + \beta y\bigr)^{\sigma - \frac{1}{p}} \int_{ - \infty}^{\infty} \frac{f(x)}{[1 + ( \vert x \vert + \alpha x)( \vert y \vert + \beta y)]^{\lambda}} \,dx\biggr\} \bigl[ \bigl( \vert y \vert + \beta y\bigr)^{\frac{1}{p} - \sigma} g(y)\bigr]\,dy \\ &\le J\biggl[ \int_{ - \infty}^{\infty} \bigl( \vert y \vert + \beta y \bigr)^{q(1 - \sigma ) - 1} g^{q}(y)\,dy\biggr]^{\frac{1}{q}}, \end{aligned} $$

and then by (12) we have (15).

On the other hand, suppose that (15) is valid. We set

$$g(y): = \bigl( \vert y \vert + \beta y\bigr)^{p\sigma - 1}\biggl\{ \int_{ - \infty}^{\infty} \frac{f(x)}{[1 + ( \vert x \vert + \alpha x)^{\delta} ( \vert y \vert + \beta y)]^{\lambda}} \,dx\biggr\} ^{p - 1}\quad (y \in \textbf{R}). $$

By (14) and the assumptions, we find $J < \infty $. If $J = 0 $, then (12) is trivially valid; if $J > 0 $, then by (15) we obtain

$$\begin{aligned}& \begin{aligned} 0 &< \int_{ - \infty}^{\infty} \bigl( \vert y \vert + \beta y \bigr)^{q(1 - \sigma ) - 1} g^{q}(y)\,dy = J^{p} = I \\ & < K(\sigma )\biggl[ \int_{ - \infty}^{\infty} \bigl( \vert x \vert + \alpha x \bigr)^{p(1 - \delta \sigma ) - 1} f^{p}(x)\,dx\biggr]^{\frac{1}{p}} \\ &\quad {}\times \biggl[ \int_{ - \infty}^{\infty} \bigl( \vert y \vert + \beta y \bigr)^{q(1 - \sigma ) - 1} g^{q}(y)\,dy\biggr]^{\frac{1}{q}} \\ &< \infty, \end{aligned} \\& \begin{aligned} J &= \biggl[ \int_{ - \infty}^{\infty} \bigl( \vert y \vert + \beta y \bigr)^{q(1 - \sigma ) - 1} g^{q}(y)\,dy\biggr]^{\frac{1}{p}} \\ &< K(\sigma ) \biggl[ \int_{ - \infty}^{\infty} \bigl( \vert x \vert + \alpha x \bigr)^{p(1 - \delta \sigma ) - 1} f^{p}(x)\,dx\biggr]^{\frac{1}{p}}. \end{aligned} \end{aligned}$$

Hence, we have (12), which is equivalent to (15).

For $n \in \textbf{N} = \{ 1,2, \ldots \}$, $n > \frac{1}{q\mu} $, we define the sets $E_{\delta}: = \{ x \in \textbf{R};|x|^{\delta} \ge 1\} $,

$$E_{\delta}^{ +}: = \bigl\{ x \in \textbf{R}_{ +};x^{\delta} \ge 1\bigr\} ,\qquad E_{\delta}^{ -}: = \bigl\{ - x \in \textbf{R}_{ +};( - x)^{\delta} \ge 1\bigr\} , $$

and the following functions:

$$\begin{aligned}& \tilde{f}(x): = \textstyle\begin{cases} ( \vert x \vert + \alpha x)^{\delta (\sigma - \frac{1}{np}) - 1},&x \in E_{\delta}, \\ 0,&x \in \textbf{R}\backslash E_{\delta}, \end{cases}\displaystyle \\& \tilde{g}(y): = \textstyle\begin{cases} ( \vert y \vert + \beta y)^{\sigma + \frac{1}{nq} - 1},&y \in [ - 1,1], \\ 0,&y \in ( - \infty, - 1) \cup (1,\infty ). \end{cases}\displaystyle \end{aligned}$$

Then we obtain that

$$\begin{aligned}& \begin{aligned} &\int_{E_{\delta}} \bigl( \vert x \vert + \alpha x \bigr)^{ - \frac{\delta}{n} - 1} \,dx\\ &\quad = \int_{E_{\delta}^{ -}} ( - x + \alpha x)^{ - \frac{\delta}{n} - 1} \,dx + \int_{E_{\delta}^{ +}} (x + \alpha x)^{ - \frac{\delta}{n} - 1} \,dx \\ &\quad = \bigl[(1 - \alpha )^{ - \frac{\delta}{n} - 1} + (1 + \alpha )^{ - \frac{\delta}{n} - 1}\bigr] \int_{E_{\delta}^{ +}} x^{ - \frac{\delta}{n} - 1} \,dx \\ &\quad = \bigl[(1 - \alpha )^{ - \frac{\delta}{n} - 1} + (1 + \alpha )^{ - \frac{\delta}{n} - 1}\bigr] \int_{1}^{\infty} \bigl(u^{\delta^{ - 1}} \bigr)^{ - \frac{\delta}{n} - 1} \bigl\vert \delta^{ - 1} \bigr\vert u^{\delta^{ - 1} - 1}\,du\quad \bigl(u = x^{\delta} \bigr) \\ &\quad = \bigl[(1 - \alpha )^{ - \frac{\delta}{n} - 1} + (1 + \alpha )^{ - \frac{\delta}{n} - 1}\bigr] \int_{1}^{\infty} u^{ - \frac{1}{n} - 1} \,du = \bigl[(1 - \alpha )^{ - \frac{\delta}{n} - 1} + (1 + \alpha )^{ - \frac{\delta}{n} - 1}\bigr]n, \end{aligned} \\& \begin{aligned} &\int_{ - 1}^{1} \bigl( \vert y \vert + \beta y \bigr)^{\frac{1}{n} - 1}\,dy\\ &\quad = \int_{ - 1}^{0} ( - y + \beta y)^{\frac{1}{n} - 1}\,dy + \int_{0}^{1} (y + \beta y)^{\frac{1}{n} - 1}\,dy \\ &\quad = \bigl[(1 - \beta )^{\frac{1}{n} - 1} + (1 + \beta )^{\frac{1}{n} - 1}\bigr] \int_{0}^{1} y^{\frac{1}{n} - 1} \,dy = \bigl[(1 - \beta )^{\frac{1}{n} - 1} + (1 + \beta )^{\frac{1}{n} - 1}\bigr]n, \end{aligned} \\& \begin{aligned} \tilde{L}&: = \biggl[ \int_{ - \infty}^{\infty} \bigl( \vert x \vert + \alpha x \bigr)^{p(1 - \delta \sigma ) - 1} \tilde{f}^{p}(x)\,dx\biggr]^{\frac{1}{p}}\biggl[ \int_{ - \infty}^{\infty} \bigl( \vert y \vert + \beta y \bigr)^{q(1 - \sigma ) - 1} \tilde{g}^{q}(y)\,dy\biggr]^{\frac{1}{q}} \\ &= \biggl[ \int_{E_{\delta}} \bigl( \vert x \vert + \alpha x \bigr)^{ - \frac{\delta}{n} - 1} \,dx\biggr]^{\frac{1}{p}}\biggl[ \int_{ - 1}^{1} \bigl( \vert y \vert + \beta y \bigr)^{\frac{1}{n} - 1}\,dy \biggr]^{\frac{1}{q}} \\ &= n\bigl[(1 - \alpha )^{ - \frac{\delta}{n} - 1} + (1 + \alpha )^{ - \frac{\delta}{n} - 1} \bigr]^{\frac{1}{p}}\bigl[(1 - \beta )^{\frac{1}{n} - 1} + (1 + \beta )^{\frac{1}{n} - 1}\bigr]^{\frac{1}{q}}. \end{aligned} \end{aligned}$$

In the same way, we still find that

$$\begin{aligned}& \int_{E_{\delta}} \bigl( \vert x \vert + \alpha x \bigr)^{ - \delta (\mu + \frac{1}{np}) - 1} \,dx = \bigl[(1 - \alpha )^{ - \delta (\mu + \frac{1}{np}) - 1} + (1 + \alpha )^{ - \delta (\mu + \frac{1}{np}) - 1}\bigr]\frac{1}{\mu + \frac{1}{np}}, \\& \int_{\textbf{R}\backslash [ - 1,1]} \bigl( \vert y \vert + \beta y \bigr)^{ - (\mu - \frac{1}{nq}) - 1}\,dy = \bigl[(1 - \beta )^{ - (\mu - \frac{1}{nq}) - 1} + (1 + \beta )^{ - (\mu - \frac{1}{nq}) - 1}\bigr] \frac{1}{\mu - \frac{1}{nq}}. \end{aligned}$$

Hence, we obtain

$$\begin{aligned} \tilde{I}&: = \int_{ - \infty}^{\infty} \int_{ - \infty}^{\infty} \frac{\tilde{f}(x)\tilde{g}(y)}{[1 + ( \vert x \vert + \alpha x)^{\delta} ( \vert y \vert + \beta y)]^{\lambda}} \,dy\,dx \\ &= \int_{E_{\delta}} \biggl[ \int_{ - 1}^{1} \frac{( \vert x \vert + \alpha x)^{\delta (\sigma - \frac{1}{np}) - 1}( \vert y \vert + \beta y)^{(\sigma + \frac{1}{nq}) - 1}}{[1 + ( \vert x \vert + \alpha x)^{\delta} ( \vert y \vert + \beta y)]^{\lambda}} \,dy\biggr]\,dx \\ &= \int_{E_{\delta}} \bigl( \vert x \vert + \alpha x \bigr)^{\frac{ - \delta}{n} - 1}\varpi_{\delta} \biggl(\sigma + \frac{1}{nq},x \biggr) \,dx \\ &\quad {}- \int_{E_{\delta}} \biggl[ \int_{\textbf{R}\backslash [ - 1,1]} \frac{( \vert x \vert + \alpha x)^{\delta (\sigma - \frac{1}{np}) - 1}( \vert y \vert + \beta y)^{\sigma + \frac{1}{nq} - 1}}{[1 + ( \vert x \vert + \alpha x)^{\delta} ( \vert y \vert + \beta y)]^{\lambda}} \,dy\biggr]\,dx \\ & \ge \int_{E_{\delta}} \bigl( \vert x \vert + \alpha x \bigr)^{\frac{ - \delta}{n} - 1}\varpi_{\delta} \biggl(\sigma + \frac{1}{nq},x \biggr) \,dx \\ &\quad {}- \int_{E_{\delta}} \biggl[ \int_{\textbf{R}\backslash [ - 1,1]} \frac{( \vert x \vert + \alpha x)^{\delta (\sigma - \frac{1}{np}) - 1}( \vert y \vert + \beta y)^{\sigma + \frac{1}{nq} - 1}}{[( \vert x \vert + \alpha x)^{\delta} ( \vert y \vert + \beta y)]^{\lambda}} \,dy\biggr]\,dx \\ & = K_{\beta} \biggl(\sigma + \frac{1}{nq}\biggr) \int_{E_{\delta}} \bigl( \vert x \vert + \alpha x \bigr)^{\frac{ - \delta}{n} - 1} \,dx \\ &\quad {}- \int_{E_{\delta}} \bigl( \vert x \vert + \alpha x \bigr)^{ - \delta (\mu + \frac{1}{np}) - 1} \,dx \int_{\textbf{R}\backslash [ - 1,1]} \bigl( \vert y \vert + \beta y \bigr)^{ - (\mu - \frac{1}{nq}) - 1}\,dy \\ & = K_{\beta} \biggl(\sigma + \frac{1}{nq}\biggr)\bigl[(1 - \alpha )^{\frac{ - \delta}{n} - 1} + (1 + \alpha )^{\frac{ - \delta}{n} - 1}\bigr]n - O(1). \end{aligned}$$

If there exists a constant $k \le K(\sigma ) $ such that (15) is valid when replacing $K(\sigma ) $ by *k*, then in particular, we have

$$\frac{1}{n}\tilde{I} = \frac{1}{n} \int_{ - \infty}^{\infty} \int_{ - \infty}^{\infty} \frac{\tilde{f}(x)\tilde{g}(y)}{[1 + ( \vert x \vert + \alpha x)^{\delta} ( \vert y \vert + \beta y)]^{\lambda}} \,dy\,dx < \frac{k}{n}\tilde{L}. $$

In view of the above results, it follows that

19 $$ \begin{gathered}[b] K_{\beta} \biggl(\sigma + \frac{1}{nq}\biggr) \bigl[(1 - \alpha )^{\frac{ - \delta}{n} - 1} + (1 + \alpha )^{\frac{ - \delta}{n} - 1}\bigr] - \frac{1}{n}O \\ \quad < k\bigl[(1 - \alpha )^{ - \frac{\delta}{n} - 1} + (1 + \alpha )^{ - \frac{\delta}{n} - 1} \bigr]^{\frac{1}{p}}\bigl[(1 - \beta )^{\frac{1}{n} - 1} + (1 + \beta )^{\frac{1}{n} - 1}\bigr]^{\frac{1}{q}}. \end{gathered} $$

For $n \to \infty $, we find

$$\frac{4B(\mu,\sigma )}{(1 - \alpha^{2})(1 - \beta^{2})} \le 2k\biggl(\frac{1}{1 - \alpha^{2}}\biggr)^{\frac{1}{p}}\biggl( \frac{1}{1 - \beta^{2}}\biggr)^{\frac{1}{q}}, $$

namely $K(\sigma ) \le k $. Hence, $k = K(\sigma ) $ is the best possible constant factor of (15).

The constant factor $K(\sigma ) $ in (12) is also the best possible. Otherwise, we can conclude a contradiction by (18) that the constant factor in (17) is not the best possible.

The theorem is proved. □

### Theorem 3

*With regards to the assumptions of Theorem *2, *replacing* $p > 1 $ *by* $0 < p < 1 $, *we have the equivalent reverses of* (12) *and* (15) *with the best possible constant factor* $K(\sigma ) $.

### Proof

We only prove that the constant factor $K(\sigma ) $ in the reverse of (15) is the best possible, and omit the others. If there exists a constant $k \ge K(\sigma ) $ such that the reverse of (15) is valid when replacing $K(\sigma ) $ by *k*, then in particular, for $n \in \textbf{N} = \{ 1,2, \ldots \}$, $n > \frac{1}{|q|\sigma} $, we have

$$\begin{gathered} k\bigl[(1 - \alpha )^{ - \frac{\delta}{n} - 1} + (1 + \alpha )^{ - \frac{\delta}{n} - 1}\bigr]^{\frac{1}{p}}\bigl[(1 - \beta )^{\frac{1}{n} - 1} + (1 + \beta )^{\frac{1}{n} - 1}\bigr]^{\frac{1}{q}} \\ \quad = \frac{k}{n}\tilde{L} < \frac{1}{n}\tilde{I} = \frac{1}{n} \int_{ - \infty}^{\infty} \int_{ - \infty}^{\infty} \frac{\tilde{f}(x)\tilde{g}(y)}{[1 + ( \vert x \vert + \alpha x)^{\delta} ( \vert y \vert + \beta y)]^{\lambda}} \,dy\,dx \\ \quad \le \frac{1}{n} \int_{E_{\delta}} \bigl( \vert x \vert + \alpha x \bigr)^{\frac{ - \delta}{n} - 1}\varpi_{\delta} \biggl(\sigma + \frac{1}{nq},x \biggr) \,dx \\ \quad = K_{\beta} \biggl(\sigma + \frac{1}{nq}\biggr)\bigl[(1 - \alpha )^{\frac{ - \delta}{n} - 1} + (1 + \alpha )^{\frac{ - \delta}{n} - 1}\bigr]. \end{gathered} $$

For $n \to \infty $, we obtain that $k \le K(\sigma ) $. Hence, $k = K(\sigma ) $ is the best possible constant factor of the reverse of (15).

The theorem is proved. □

## Operator expressions and a remark

For $p > 1$, $\frac{1}{p} + \frac{1}{q} = 1$, $\delta \in \{ - 1,1\}$, $- 1 < \alpha,\beta < 1$, $\mu,\sigma > 0$, $\mu + \sigma = \lambda $, we set the following functions: $\varphi (x): = (|x| + \alpha x)^{p(1 - \delta \sigma ) - 1}$, $\psi (y): = (|y| + \beta y)^{q(1 - \sigma ) - 1}$, where from

$$\psi^{1 - p}(y) = \bigl( \vert y \vert + \beta y\bigr)^{p\sigma - 1} \quad (x,y \in \textbf{R}). $$

Define the following real normed linear spaces:

$$\begin{aligned}& L_{p,\varphi} (\textbf{R}): = \biggl\{ f; \Vert f \Vert _{p,\varphi}: = \biggl( \int_{ - \infty}^{\infty} \varphi (x) \bigl\vert f(x) \bigr\vert ^{p} \,dx\biggr)^{\frac{1}{p}} < \infty \biggr\} , \\& L_{p,\psi^{1 - p}}(\textbf{R}): = \biggl\{ h; \Vert h \Vert _{p,\psi^{1 - p}}: = \biggl( \int_{ - \infty}^{\infty} \psi^{1 - p}(y) \bigl\vert h(y) \bigr\vert ^{p} \,dy\biggr)^{\frac{1}{p}} < \infty \biggr\} , \\& L_{q,\psi} (\textbf{R}): = \biggl\{ g; \Vert g \Vert _{q,\psi}: = \biggl( \int_{ - \infty}^{\infty} \psi (y) \bigl\vert g(y) \bigr\vert ^{q} \,dy\biggr)^{\frac{1}{q}} < \infty \biggr\} . \end{aligned}$$

In view of Theorem 1, for any $f \in L_{p,\varphi} (\textbf{R}) $, we set

$$h(y): = \int_{ - \infty}^{\infty} \frac{1}{[1 + ( \vert x \vert + \alpha x)^{\delta} ( \vert y \vert + \beta y)]^{\lambda}} f(x)\,dx \quad (y \in \textbf{R}). $$

By (12) we have

20 $$ \Vert h \Vert _{p,\psi^{1 - p}} = \biggl( \int_{ - \infty}^{\infty} \psi^{1 - p}(y) \bigl\vert h(y) \bigr\vert ^{p} \,dy\biggr)^{\frac{1}{p}} \le K(\sigma ) \Vert f \Vert _{p,\varphi} < \infty. $$

### Definition 2

Define an extended Hilbert’s integral operator in the whole plane

$$T:L_{p,\varphi} (\textbf{R}) \to L_{p,\psi^{1 - p}}(\textbf{R}) $$

as follows: For any $f \in L_{p,\varphi} (\textbf{R}) $, there exists $Tf = h \in L_{p,\psi^{1 - p}}(\textbf{R}) $.

In view of (20), the operator *T* is bounded with

$$\Vert T \Vert = \sup_{f( \ne \theta ) \in L_{p,\varphi} (\textbf{R})}\frac{ \Vert Tf \Vert _{p,\psi^{1 - p}}}{ \Vert f \Vert _{p,\varphi}} \le K(\sigma ). $$

Since by Theorem 2 the constant factor in (20) is the best possible, we have

21 $$ \Vert T \Vert = K(\sigma ) = \frac{2B(\mu,\sigma )}{(1 - \beta^{2})^{1/p}(1 - \alpha^{2})^{1/q}}. $$

If we define the normal inner product of *Tf* and *g* as follows:

$$(Tf,g): = \int_{ - \infty}^{\infty} \biggl\{ \int_{ - \infty}^{\infty} \frac{1}{[1 + ( \vert x \vert + \alpha x)^{\delta} ( \vert y \vert + \beta y)]^{\lambda}} f(x)\,dx\biggr\} g(y)\,dy, $$

then we can rewrite (15) and (12) as the following equivalent operator expressions:

22 $$ (Tf,g) < \Vert T \Vert \cdot \Vert f \Vert _{p,\varphi} \Vert g \Vert _{q,\psi},\qquad \Vert Tf \Vert _{p,\psi^{1 - p}} < \Vert T \Vert \cdot \Vert f \Vert _{p,\varphi}. $$

### Remark 1

(i) In Theorem 2, for $\delta = - 1 $, replacing $(|x| + \alpha x)^{\lambda} f(x) $ by $f(x) $, we have

$$0 < \int_{ - \infty}^{\infty} \bigl( \vert x \vert + \alpha x \bigr)^{p(1 - \mu ) - 1} f^{p}(x)\,dx < \infty, $$

and the following equivalent inequalities with the homogeneous kernel and the best possible constant factor $K(\sigma ) = \frac{2B(\mu,\sigma )}{(1 - \beta^{2})^{1/p}(1 - \alpha^{2})^{1/q}} $:

23 $$\begin{aligned}& \begin{gathered}[b] \biggl\{ \int_{ - \infty}^{\infty} \bigl( \vert y \vert + \beta y \bigr)^{p\sigma - 1}\biggl[ \int_{ - \infty}^{\infty} \frac{f(x)}{( \vert x \vert + \alpha x + \vert y \vert + \beta y)^{\lambda}} \,dx \biggr]^{p}\,dy\biggr\} ^{\frac{1}{p}} \\ \quad < \frac{2B(\mu,\sigma )}{(1 - \beta^{2})^{1/p}(1 - \alpha^{2})^{1/q}}\biggl[ \int_{ - \infty}^{\infty} \bigl( \vert x \vert + \alpha x \bigr)^{p(1 - \mu ) - 1} f^{p}(x)\,dx\biggr]^{\frac{1}{p}}, \end{gathered} \end{aligned}$$

24 $$\begin{aligned}& \begin{gathered}[b] \int_{ - \infty}^{\infty} \int_{ - \infty}^{\infty} \frac{f(x)g(y)}{( \vert x \vert + \alpha x + \vert y \vert + \beta y)^{\lambda}} \,dx \,dy \\ \quad < \frac{2B(\mu,\sigma )}{(1 - \beta^{2})^{1/p}(1 - \alpha^{2})^{1/q}}\biggl[ \int_{ - \infty}^{\infty} \bigl( \vert x \vert + \alpha x \bigr)^{p(1 - \mu ) - 1} f^{p}(x)\,dx\biggr]^{\frac{1}{p}} \\ \qquad {}\times \biggl[ \int_{ - \infty}^{\infty} \bigl( \vert y \vert + \beta y \bigr)^{q(1 - \sigma ) - 1} g^{q}(y)\,dy\biggr]^{\frac{1}{q}}. \end{gathered} \end{aligned}$$

(ii) For $\alpha = \beta = 0 $, inequality (24) reduces to (6), and (17) reduces to

25 $$ \begin{gathered}[b] \int_{ - \infty}^{\infty} \int_{ - \infty}^{\infty} \frac{f(x)g(y)}{(1 + \vert xy \vert )^{\lambda}} \,dx \,dy \\ \quad < 2B(\mu,\sigma )\biggl[ \int_{ - \infty}^{\infty} \vert x \vert ^{p(1 - \sigma ) - 1} f^{p}(x)\,dx\biggr]^{\frac{1}{p}}\biggl[ \int_{ - \infty}^{\infty} \vert y \vert ^{q(1 - \sigma ) - 1} g^{q}(y)\,dy\biggr]^{\frac{1}{q}}. \end{gathered} $$

(iii) For $p = q = 2$, $\mu = \sigma = \frac{\lambda}{2}$, $f( - x) = f(x)$, $g( - y) = g(y) $ ($x,y > 0 $), inequality (6) reduces to (4). Hence, inequality (6) is an extended Hilbert’s integral inequality in the whole plane, and inequality (15) is a more general form of (6) with parameters.

## Conclusions

In this paper, by introducing independent parameters and interval variables, applying the weight functions and the technique of real analysis, an extended Hilbert’s integral inequality in the whole plane with parameters and a best possible constant factor is provided in Theorem 2. The equivalent forms, the reverses, and the related homogeneous forms with particular parameters are considered. An extended Hilbert’s integral operator in the whole plane is defined, and the operator expressions for the equivalent inequalities are obtained. The method of weight functions is very important, which helps us to prove the equivalent inequalities with the best possible constant factor. The lemmas and theorems provide an extensive account of this type of inequalities.
